# Deriving national and disaggregated estimates for the demand for family planning satisfied indicator from contraceptive prevalence using household health surveys

**DOI:** 10.1186/s12978-025-02187-8

**Published:** 2025-11-12

**Authors:** Leonardo Z. Ferreira, Franciele Hellwig, Natália P. Lima, Aluísio J. D. Barros

**Affiliations:** International Center for Equity in Health, Post-Graduate Program in Epidemiology, Federal University of Pelotas, R. Marechal Deodoro 1160, Centro, Pelotas, Brazil

**Keywords:** Family planning, Contraceptives, Regression models, Health surveys, Reproductive health

## Abstract

**Background:**

Demand for family planning satisfied (DFPS) is one of the core indicators for monitoring reproductive health. However, it involves a series of questions that are not always available in national health surveys, hindering the international comparability and tracking progress in those settings. This study updates an alternative method for calculating DFPS based on contraceptive prevalence (CPR) when direct estimation is not feasible.

**Methods:**

Based on survey data from 1,099 subnational regions across 103 countries, we fitted least-squares regression models that predicts DFPS for both any contraceptive methods and modern methods. A fractional polynomial approach was employed to account for non-linear relationships. Model performance was assessed using a 5-fold cross-validation strategy, evaluating bias, mean absolute error and correlation.

**Results:**

The models, using CPR and the difference between total and modern CPR as predictors, were able to explain over 97% of the variability of DFPS by any and modern contraceptives. Bias and the magnitude of the errors were around 0.1 in the cross-validated sample. Validating the results for other inequality dimensions beyond subnational region yielded even better metrics.

**Conclusions:**

The predicted estimates proved to be a good approximation for DFPS in circumstances where direct estimation is not possible. This study confirms that modeling DFPS through contraceptive prevalence remains valid and extends its applicability to DFPS based on modern methods. Furthermore, the estimates were robust for varying inequality dimensions, allowing equity analyses to be performed when appropriate.

**Supplementary Information:**

The online version contains supplementary material available at 10.1186/s12978-025-02187-8.

## Introduction

Access to sexual and reproductive health is a critical component of overall health and well-being and a recognized human right. It encompasses a range of issues, including access to information and services related to family planning. Demand for family planning satisfied by modern methods (DFPSm) is a key indicator for monitoring sexual and reproductive health globally and achieving the Sustainable Development Goals [1, 2]. This indicator represents the proportion of women in need of contraception who are using a modern method to avoid unintended pregnancies. However, measuring this indicator in household health surveys can be challenging as it requires collecting information on several questions related to contraceptive use, method type, women’s fertility, and fertility intentions. Although it is already available in most Demographic and Health Surveys (DHS) and Multiple Indicator Cluster Surveys (MICS), national health surveys outside these programs often do not collect the necessary information.

Statistical models built to estimate health indicators, including family planning, have long been established in the literature. Researchers developed a Bayesian hierarchical model to produce annual estimates for married or in-union women of reproductive age [3, 4]. This approach was later expanded to include unmarried women and the prevalence of sexual activity within this group across countries [5, 6]. Building on this methodology, the Family Planning Estimation Tool (FPET) was created, enabling users to generate annual family planning estimates while allowing users to tune specific parameters and incorporate service statistics when available [7]. While these sophisticated Bayesian models provide robust estimates, even within a user-friendly tool, they still require specialized training, understanding of the underlying assumptions and careful interpretation in certain contexts. Finally, Bayesian models are often designed to provide national-level estimates only, limiting their applicability for equity analyses.

In 2015, Barros and colleagues [8] proposed an alternative approach to obtain estimates of demand for family planning satisfied by any contraceptive method (DFPSany), using a linear regression model with a logit-transformed outcome based on contraceptive prevalence, in situations where it cannot be estimated directly. Due to the dependence of traditional methods on their correct usage and a greater degree of planning and self-control, they are increasingly becoming less utilized [9]. As the SDG indicator for tracking access to family planning focuses on modern methods, we propose an updated version of the original approach, tailored to estimate DFPSm. Additionally, in 2012, changes were proposed to enhance the calculation of the need for contraception and make it consistent over time and across different surveys. These changes involved standardizing questions used by different international survey programs, addressing inconsistencies and missing information, shortening the duration of which women are considered postpartum amenorrheic, and refining the calculation of infecundity [10]. Thus, we validate the method by incorporating the latest available data based on the revised definition.

The lack of necessary information to estimate demand for family planning satisfied (DFPS) poses a significant challenge for academics, policymakers, and health professionals in tracking progress in access to sexual and reproductive health. Without a reliable indicator, incorrect conclusions about progress or lack thereof could be drawn, hindering effective policymaking. This paper aims to provide a stable method for estimating the demand for family planning satisfied by modern methods in the absence of sufficient information for identifying women in need of contraception. Our study provides an updated analysis of Barros and colleagues’ [8] work by utilizing the most recent data points for all countries, using the revised definition of unmet need for contraception, and focusing on modern contraceptives. We also extend the model’s applicability by validating it in multiple levels, representing different inequality dimensions (national, subnational, area of residence, wealth quintiles, and woman’s education).

## Methods

We used data from the most recent national health survey from low- and middle-income countries with available information on family planning. The surveys use standardized questionnaires to collect data from reproductive-aged women and young children selected using multi-stage sampling procedures. Generally, all women aged 15 to 49 are eligible to be interviewed in the survey, but some countries restrict the sampling to ever-married women. To include a larger number of countries and ensure comparability, only women married or in a union were included in our analysis.

We calculated four family planning indicators, including contraceptive prevalence by any methods (CPRany), contraceptive prevalence by modern methods (CPRm), DFPSany, and DFPSm. All the indicators share the same numerator - the number of partnered women 15–49 years using contraceptives (any or modern, depending on the indicator). The denominator for CPRany and CPRm is partnered women aged 15–49 years, while the denominator for DFPSany and DFPSm includes only those women in need of contraception. Fecund women who do not desire a child in the next two years or wish to delay pregnancy are considered in need. Women who are pregnant or in postpartum amenorrhea whose pregnancy was undesired are also considered in need of contraception. Infecund women (those who fall in any of the following situations: (i) never menstruated; (ii) are menopausal; (iii) do not have an under-five child and the last period was more than six months ago; (iv) are married and did not have a child while not using contraception in the past five years and; (v) declared that are unable to conceive) are not considered in need of contraception. More details on the indicator definitions are found elsewhere [10].

Modern contraceptive methods were defined according to Hubacher and Trussell’s [11]. Their definition of modern contraceptives includes condoms, sterilization (male and female), intrauterine devices, implants, oral contraceptives, injectables, emergency pills, patches, diaphragms, spermicidal agents, vaginal rings, and sponges.

We generated estimates for all four indicators at five levels (national, subnational, area of residence, wealth quintiles, and woman’s education) from all available surveys. These estimates were pooled into five separate datasets, one for each inequality dimension. Sampling weights and the clustered design of the surveys were accounted in the calculation of the estimates.

For each family planning indicator (DFPSany and DFPSm), we fitted separate linear regression models across each of the five inequality dimensions, resulting in a total of ten models. Within each of these ten, we tested multiple model specifications, exploring a combination of predictors and the use of fractional polynomials to account for non-linear relationships. In the end, all models yielded very similar results. Therefore, for each indicator, we selected and present a single model — the one based on subnational regions — as it comprised more units of analysis and better reflected diverse contexts.

Prior to model fitting, we applied a logit transformation to the DFPS indicators to ensure predicted values remained within the [0–1] interval. The transformed values served as the outcome variable in linear regression models. The primary predictor was the contraceptive prevalence rate (for any or modern methods, accordingly), while other potential predictors considered included the difference between CPRany and CPRm, the ratio between CPRany and CPRm, and the total fertility rate. Fractional polynomials of up to the second order were tested to capture potential non-linear relationships. Contraceptive prevalence rates below 1% were excluded from the analysis to mitigate potential instability in the models. The hierarchy of subnational regions nested within each country was accounted when calculating the standard errors in the adjustment of the model at this level.

To evaluate the models’ predictive performance, we used a 5-fold cross-validation strategy which involves dividing the data into five subsets, training the models on four subsets, and evaluating their performance on the remaining subset. Subsets are cycled until all of them are used for training and validation. The out-of-sample accuracy of the predictions was quantified by computing bias, mean absolute error and correlation metrics. Predicted DFPS values were back-transformed to the original scale for interpretation purposes.

Finally, sensitivity analyses were tested by restricting the sample for contraceptive rates of 15% or above based on the slope of the regression in the original publication. All analyses were carried out using Stata version 17 [12].

## Results

Estimates for all indicators were obtained from 103 countries (47 DHS, 55 MICS and 1 country-specific survey), with the survey year ranging from 2009 to 2021. This allowed estimates for 1,099 subnational regions. The summary statistics of the four indicators under study are presented in Table 1. On average, DFPSany and DFPSm estimates are around 20% points (p.p) higher than CPRm and CPRany. All indicators varied widely between settings with DFPSm ranging from 6.0% to 87.9% and CPRm ranging from 3.7% to 75.8%.

**Table 1:** Summary statistics of the four family planning indicators for the subnational regions in the sample

Indicator	Mean	Minimum	10th percentile	Median	90th percentile	Maximum
DFPSany	62.9%	4.7%	29.1%	68.0%	88.0%	96.4%
DFPSm	52.9%	2.8%	19.4%	55.3%	82.6%	94.2%
CPRany	41.8%	1.5%	11.8%	43.7%	69.3%	84.8%
CPRm	35.1%	1.2%	8.3%	34.3%	64.1%	84.8%
CPRany – CPRm (p.p.)	6.7	0.0	0.4	3.4	16.4	63.9

*DFPSany* Demand for family planning satisfied by any methods, *CPRany* Contraceptive prevalence by any methods, *DFPSm* Demand for family planning satisfied by modern methods, *CPRm* Contraceptive prevalence by modern methods

For DFPSany, the best fractional polynomial model used the log of CPRany, CPRany to the second power and the difference between CPRany and CPRm (*cpdiff*) as predictors, and can be described as follows:

$$\begin{aligned}\:logit\left(DFPSany\right)=&1.05+\left(\text{log}\left(CPRany\right)*0.93\right)\\&+\left(CP{Rany}^{2}*2.49\right)\:\\&+\left(cpdiff*\:0.70\right)\end{aligned}$$

Similarly, the same model specification performed best for DFPSm, resulting in the following equation:

$$\begin{aligned}\:logit\left(DFPSm\right)=&1.12+\left(\text{log}\left(CPRm\right)*0.97\right)\\&+\left(CP{Rm}^{2}*2.13\right)\\&+(cpdiff*\:-1.43)\end{aligned}$$

*Cpdiff* was included in the model as it provided a better fit than the ratio between CPRany and CPRm while total fertility rate was not significant.

Table 2 presents the estimated coefficients, p-values, and confidence intervals. All predictors in both models were highly significant (*p* < 0.001). The positive coefficient for cpdiff in the DFPSany model suggests that as the gap between any and modern contraceptive use increases (i.e., more reliance on traditional methods), DFPSany increases—likely reflecting that traditional methods still contribute to meeting family planning needs in that context. In contrast, the negative coefficient for cpdiff in the DFPSm model indicates that a larger gap between any and modern methods is associated with lower DFPSm, which aligns with expectations: greater reliance on traditional methods lowers the proportion of demand satisfied by modern methods.

**Table 2:** – Fractional polynomial model for predicting demand for family planning satisfied indicators from contraceptive prevalence indicators

Variable	Coefficient	*p*	95% CI	
DFPSany				
Intercept	1.05	< 0.001	0.88	1.22
Log(CPRany)	0.93	< 0.001	0.84	1.02
CPRany²	2.49	< 0.001	2.14	2.83
*Cpdiff*	0.70	< 0.001	0.37	1.03
DFPSm				
Intercept	1.12	< 0.001	1.00	1.25
Log(CPRm)	0.97	< 0.001	0.91	1.02
CPRm²	2.13	< 0.001	1.83	2.43
*Cpdiff*	−1.43	< 0.001	−1.70	−1.15

*DFPSany* Demand for family planning satisfied by any methods, *CPRany* Contraceptive prevalence by any methods, *DFPSm* Demand for family planning satisfied by modern methods, *CPRm* Contraceptive prevalence by modern methods, *Cpdiff* CPRany – CPRm

These models were compared against the best fractional polynomial model of each of the other four inequality dimensions and differences were negligible (Supplementary Table 2), suggesting the selected model generalizes well across different population strata. We investigated the residuals of the models, and the fit was considered good (Supplementary Fig. 1). Then, we assessed the out-of-sample predictive performance of the subnational region model using cross-validated metrics and used the equations presented above to calculate the same metrics for the other four inequality dimensions. The cross-validated results were similar for DFPSany and DFPSm with an R-squared above 97%, bias around 0.1 and the magnitude of the erros ranged between 0.08 and 0.11. Out-of-sample predictions across the national level, place of residence, wealth quintiles, and women’s education showed similar or even better performance. In these cases, the R-squared exceeded 96%, bias was close to zero, and the magnitude of the errors averaged 0.04 (Table 3).

**Table 3:** – Out-of-sample 5-fold cross-validation of the selected models for all inequality dimensions

Indicator	Dimension	*R*²	Bias	MAE
Cross-validation				
DFPSany	Subnational	0.97	0.10	0.11
DFPSm	Subnational	0.98	0.07	0.08
Out-of-sample prediction				
DFPSany	National	0.97	−0.02	0.04
	Place of residence	0.97	−0.02	0.04
	Wealth quintiles	0.97	−0.02	0.04
	Woman’s education	0.96	−0.02	0.05
DFPSm	National	0.98	−0.01	0.04
	Place of residence	0.97	−0.02	0.04
	Wealth quintiles	0.97	−0.02	0.04
	Woman’s education	0.96	−0.02	0.05

*DFPSany* Demand for family planning satisfied by any methods, *DFPSm* Demand for family planning satisfied by modern methods, *MAE* Mean absolute error

**Fig. 1:**
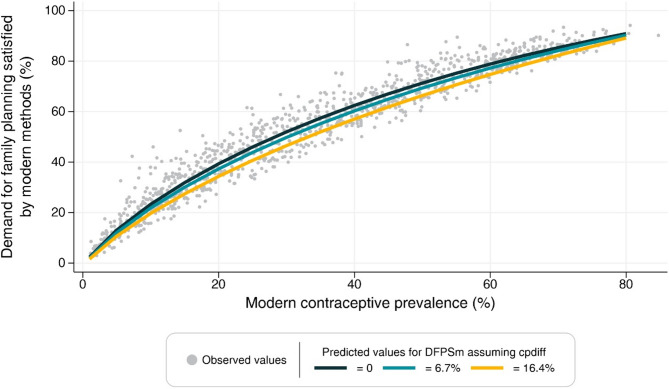
– Predicted values of DFPSm for different values of modern contraceptive prevalence and difference between total and modern contraceptive

**Fig. 2:**
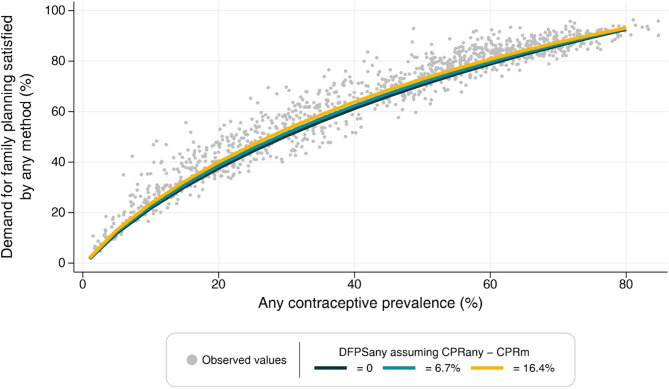
– Predicted values of DFPSany for different values of contraceptive prevalence and difference between total and modern contraceptive

Figures 1 and 2 present the estimated values for DFPSm and DFPSany for varying levels of CPRany and CPRm where each line assumes a specific value for the difference between contraceptive prevalence (10th percentile, mean and 90th percentile of the observed distribution). In the extremes, the predicted values are very similar across the slopes. However, as the difference between contraceptive prevalence increases, DFPSany and DFPSm tend to be slightly lower around the 50% mark of CPRany or CPRm. In other words, large differences between contraceptive prevalences imply a smaller share of modern contraceptive methods in the total contraceptive prevalence.

We also carried out a sensitivity analysis removing from the national level samples the estimates of CPRany or CPRm below 15%. The results confirm a linear relationship between DFPSany and CPRany (same patterns for DFPSm and CPRm) where the linear model is not statistically different from any model using fractional polynomials (Supplementary Fig. 2). While the linear model could be used in scenarios where the prevalence of CPRany is not so low and it is easier to interpret, the proposed equation also accounts for a wider range of estimates which is particularly useful in equity analyses.

## Discussion

In this study, we validated and updated the approach proposed by Barros et al. for estimating DFPSany values using a model based on CPRany. We demonstrated that this approach remains valid and applicable, even with advancements in data collection and calculation methods for DFPSany, as well as the inclusion of a decade’s worth of new data. The current modeling strategy also leverages on an additional predictor consisting of the difference between modern and any contraceptive methods use. This variable accounts for the slightly different relationships between DFPSany and CPRany, particularly in settings where the proportion of contraceptive users using a modern methods is too low. Furthermore, our findings indicate that the same modeling strategy is effective when applied to DFPSm. Additionally, we observed that the modeling approach generalizes well across various dimensions of inequality, making it suitable for equity analysis.

In our revised approach, we deviated from the original methodology by moving away from using multiple time points within each country. Instead, we utilized the most recent survey from each country as our primary data source. Notably, we made a significant change in the unit of analysis, shifting from the survey level to subnational regions within the most recent survey. This adjustment was advantageous as it reduced the potential bias arising from countries with multiple time points and introduced a greater diversity of contexts due to the inherent heterogeneity of subnational regions. Moreover, to assess the predictive performance of our model, we implemented an out-of-sample validation procedure. This validation involved testing the model on data points not included in the training set, allowing us to evaluate its generalizability and calculate metrics to quantitatively evaluate the model’s predictive efficiency.

Compared to the precision typically achieved through direct estimates from survey data, it is evident that the magnitude of error in the predicted estimates may appear relatively large. While more sophisticated approaches, such as Bayesian hierarchical models, do not fully match the accuracy of direct measurement, they generally produce more precise and robust estimates than our proposed model. In contrast, our approach is intentionally simpler, designed to generate indirect estimates from a simple yet effective model – one that is accessible, ready-to-use and generalizable across multiple countries and various inequality dimensions. It does not aim to outperform complex Bayesian methods but offers a practical and valuable alternative in contexts where such models are not feasible or where usability is key.

Some countries, especially in Asia and Northern Africa, do not collect information on contraceptive practices for unmarried women. This restriction can be attributed to strong cultural beliefs that prevail in certain countries. The emphasis on partnered women limits our understanding of the needs and behaviors of all women of reproductive age. Yet, in order to maintain the comparability between all settings, our study restricted the indicators to women married or in union. Still, we believe that the proposed method should generalize well for the indicator that includes all women, provided that the pattern of contraceptive use among married and unmarried women is similar within a given country. Nevertheless, further analysis is warranted to extend the method and verify the applicability of the approach, particularly considering that the relationship between contraceptive use and demand satisfied may differ in countries where the contraceptive practices among married and unmarried women diverge. Finally, the proposed methodology may not be entirely suitable for inequality dimensions such as religion and ethnicity. These dimensions encompass significant cultural aspects, including diverse fertility desires, which can influence the relationship between contraceptive use and demand satisfied and impact the estimates derived from the model.

## Conclusion

Demand for family planning satisfied continue as one of the key indicators for monitoring sexual and reproductive health, especially when estimates are derived from survey data. The strength of the indicator is its ability to capture women in need of contraception. However, to accurately identify women in need of contraception, comprehensive data collection is necessary, involving a series of questions regarding desired family size, pregnancy intentions and fecundity. While these questions are crucial for identifying the target population, they increase the burden of data collection and the complexity of its measurement. Our study provides an important alternative using a simple model that relies solely on whether the woman is using contraceptive methods. It proved to work effectively regardless of whether the indicator is based on modern or any methods and it is robust enough to provide estimates for different inequality dimensions.

## Supplementary Information

Supplementary Material 1.

## Data Availability

The original datasets are available from Demographic and Health Survey (https://dhsprogram.com/) and Multiple Indicator Cluster Survey (https://mics.unicef.org/surveys) websites upon registration. The processed datasets are available from the corresponding author on reasonable request.

## References

[1] Canning D, Schultz TP. The economic consequences of reproductive health and family planning. Lancet. 2012;380(9837):165–71. 22784535 10.1016/S0140-6736(12)60827-7

[2] Fabic MS, Choi Y, Bongaarts J, Darroch JE, Ross JA, Stover J, et al. Meeting demand for family planning within a generation: the post-2015 agenda. Lancet. 2015;385(9981):1928–31. 24993915 10.1016/S0140-6736(14)61055-2 PMC4393371

[3] Alkema L, Kantorová V, Menozzi C, Biddlecom A. National, regional, and global rates and trends in contraceptive prevalence and unmet need for family planning between 1990 and 2015: a systematic and comprehensive analysis. Lancet. 2013;381:1642–52. 23489750 10.1016/S0140-6736(12)62204-1

[4] Cahill N, Sonneveldt E, Stover J, Weinberger M, Williamson J, Wei C, et al. Modern contraceptive use, unmet need, and demand satisfied among women of reproductive age who are married or in a union in the focus countries of the family planning 2020 initiative: a systematic analysis using the family planning Estimation tool. Lancet. 2017;391:870–82. 29217374 10.1016/S0140-6736(17)33104-5 PMC5854461

[5] Wheldon MC, Kantorová V, Ueffing P, Dasgupta ANZ. Methods for estimating and projecting key family planning indicators among all women of reproductive age. Population Division. New York (NY): United Nations, Department of Economic and Social Affairs; 2018. Technical Paper No. 2.

[6] Kantorová V et al. Estimating progress towards meeting women’s contraceptive needs in 185 countries: a bayesian hierarchical modelling study. PLoS Med. 2020;17(2):e1003026. 10.1371/journal.pmed.1003026 PMC7028249 32069289

[7] Track20. Family Planning Estimation Tool (FPET). Available from: https://www.track20.org/pages/track20_tools/FPET.php. Cited 2025 Apr 10.

[8] Barros AJD, Boerma T, Hosseinpoor AR, Restrepo-Méndez MC, Wong KLM, Victora CG. Estimating family planning coverage from contraceptive prevalence using National household surveys. Glob Health Action. 2015;8(1):29735. 26562141 10.3402/gha.v8.29735 PMC4642361

[9] Bertrand JT, Ross J, Glover AL. Declining yet persistent use of traditional contraceptive methods in low- and middle-income countries. J Biosoc Sci. 2022;54(5):742–59. 34269170 10.1017/S0021932021000341 PMC11753471

[10] Bradley, SEK., Croft TN, Fishel JD, Westoff CF. Revising Unmet Need for Family Planning. DHS Analytical Studies No. 25. Maryland: ICF International; 2012.

[11] Hubacher D, Trussell J. A definition of modern contraceptive methods. Contraception. 2015;92(5):420–1. 26276245 10.1016/j.contraception.2015.08.008

[12] StataCorp. Stata statistical software: release 17. College station. (TX): StataCorp LLC; 2021.

