# Supplementary material for: Deriving national and disaggregated estimates for the demand for family planning satisfied indicator from contraceptive prevalence using household health surveys
Source: Reprod Health. 2025 Nov 12;22:228. doi: 10.1186/s12978-025-02187-8 (PMC12606838; doi:10.1186/s12978-025-02187-8)
Supplement: Supplementary file 1 — Supplementary Material 1. [file 12978_2025_2187_MOESM1_ESM.docx]

# Supplementary Materials

Supplementary Table 1 – Characteristics of the surveys included in the study

| **ISO3 country code** | **Country name** | **Year** | **Source** |
| --- | --- | --- | --- |
| AFG | Afghanistan | 2015 | DHS |
| ALB | Albania | 2017 | DHS |
| DZA | Algeria | 2018 | MICS |
| AGO | Angola | 2015 | DHS |
| ARG | Argentina | 2019 | MICS |
| ARM | Armenia | 2015 | DHS |
| BGD | Bangladesh | 2019 | MICS |
| BRB | Barbados | 2012 | MICS |
| BLR | Belarus | 2019 | MICS |
| BLZ | Belize | 2015 | MICS |
| BEN | Benin | 2021 | MICS |
| BTN | Bhutan | 2010 | MICS |
| BIH | Bosnia and Herzegovina | 2011 | MICS |
| BFA | Burkina Faso | 2021 | DHS |
| BDI | Burundi | 2016 | DHS |
| CAF | Central African Republic | 2018 | MICS |
| KHM | Cambodia | 2021 | DHS |
| CMR | Cameroon | 2018 | DHS |
| TCD | Chad | 2019 | MICS |
| COL | Colombia | 2015 | DHS |
| COM | Comoros | 2022 | MICS |
| COG | Congo, Republic of | 2014 | MICS |
| COD | Congo, Democratic Republic of the | 2017 | MICS |
| CRI | Costa Rica | 2018 | MICS |
| CIV | Côte d’Ivoire | 2021 | DHS |
| CUB | Cuba | 2019 | MICS |
| DOM | Dominican Republic | 2019 | MICS |
| ECU | Ecuador | 2018 | NSS |
| EGY | Egypt | 2014 | DHS |
| SLV | El Salvador | 2014 | MICS |
| SWZ | Eswatini | 2021 | MICS |
| ETH | Ethiopia | 2016 | DHS |
| FJI | Fiji | 2021 | MICS |
| GAB | Gabon | 2019 | DHS |
| GMB | Gambia | 2019 | DHS |
| GHA | Ghana | 2022 | DHS |
| GTM | Guatemala | 2014 | DHS |
| GIN | Guinea | 2018 | DHS |
| GNB | Guinea-Bissau | 2018 | MICS |
| GUY | Guyana | 2019 | MICS |
| HTI | Haiti | 2016 | DHS |
| HND | Honduras | 2019 | MICS |
| IND | India | 2019 | DHS |
| IDN | Indonesia | 2017 | DHS |
| IRQ | Iraq | 2018 | MICS |
| JOR | Jordan | 2017 | DHS |
| KAZ | Kazakhstan | 2015 | MICS |
| KEN | Kenya | 2022 | DHS |
| KIR | Kiribati | 2018 | MICS |
| XKX | Kosovo | 2019 | MICS |
| KGZ | Kyrgyzstan | 2018 | MICS |
| LAO | Lao People's Democratic Republic | 2017 | MICS |
| LSO | Lesotho | 2018 | MICS |
| LBR | Liberia | 2019 | DHS |
| MDG | Madagascar | 2021 | DHS |
| MWI | Malawi | 2019 | MICS |
| MDV | Maldives | 2016 | DHS |
| MLI | Mali | 2018 | DHS |
| MRT | Mauritania | 2019 | DHS |
| MEX | Mexico | 2015 | MICS |
| MDA | Moldova | 2012 | MICS |
| MNG | Mongolia | 2018 | MICS |
| MNE | Montenegro | 2018 | MICS |
| MOZ | Mozambique | 2015 | DHS |
| MMR | Myanmar | 2015 | DHS |
| NAM | Namibia | 2013 | DHS |
| NPL | Nepal | 2022 | DHS |
| NER | Niger | 2021 | DHS |
| NGA | Nigeria | 2021 | MICS |
| PAK | Pakistan | 2017 | DHS |
| PAN | Panama | 2013 | MICS |
| PNG | Papua New Guinea | 2016 | DHS |
| PRY | Paraguay | 2016 | MICS |
| PHL | Philippines | 2022 | DHS |
| QAT | Qatar | 2012 | MICS |
| RWA | Rwanda | 2019 | DHS |
| WSM | Samoa | 2019 | MICS |
| STP | Sao Tome and Principe | 2019 | MICS |
| SEN | Senegal | 2019 | DHS |
| SRB | Serbia | 2019 | MICS |
| SLE | Sierra Leone | 2019 | DHS |
| ZAF | South Africa | 2016 | DHS |
| LCA | St Lucia | 2012 | MICS |
| PSE | Palestine, State of | 2019 | MICS |
| SDN | Sudan | 2014 | MICS |
| SUR | Suriname | 2018 | MICS |
| TJK | Tajikistan | 2017 | DHS |
| TZA | Tanzania | 2022 | DHS |
| THA | Thailand | 2022 | MICS |
| TLS | Timor-Leste | 2016 | DHS |
| TGO | Togo | 2017 | MICS |
| TON | Tonga | 2019 | MICS |
| TTO | Trinidad and Tobago | 2022 | MICS |
| TUN | Tunisia | 2018 | MICS |
| TUR | Turkey | 2018 | DHS |
| TKM | Turkmenistan | 2019 | MICS |
| TCA | Turks and Caicos | 2019 | MICS |
| UGA | Uganda | 2016 | DHS |
| UKR | Ukraine | 2012 | MICS |
| UZB | Uzbekistan | 2021 | MICS |
| VNM | Vietnam | 2020 | MICS |
| YEM | Yemen | 2013 | DHS |
| ZMB | Zambia | 2018 | DHS |
| ZWE | Zimbabwe | 2015 | DHS |

Supplementary Table 2 – Comparison of best fractional polynomial model at the subnational level and the best model for each of the other four levels

| **Indicator** | **Unit of analysis** | **Best model** | | | **Best subnational model** | |
| --- | --- | --- | --- | --- | --- | --- |
|  |  | **Powers** | **Predicted correlation** | **BIC** | **Predicted correlation** | **BIC** |
|  | National | CPRany^-2^ CPRany | 0.966 | -467 | 0.966 | -467 |
|  | Place of residence | CPRany^-1^ CPRany | 0.968 | -1066 | 0.968 | -1066 |
|  | Wealth quintiles | CPRany^-1^ CPRany | 0.969 | -3190 | 0.969 | -3190 |
|  | Woman’s education | Log CPRany² | 0.962 | -1545 | 0.962 | -1545 |
|  | National | CPRm^-1^ CPRm | 0.974 | -469 | 0.974 | -469 |
|  | Place of residence | Log CPRm³ | 0.976 | -1070 | 0.976 | -1070 |
|  | Wealth quintiles | Log CPRm² | 0.977 | -3201 | 0.977 | -3201 |
|  | Woman’s education | Log CPRm³ | 0.972 | -1549 | 0.972 | -1548 |

DFPSany: Demand for family planning satisfied by any methods; CPR: contraceptive prevalence by any methods; DFPSm: Demand for family planning satisfied by modern methods; CPRm: contraceptive prevalence by modern methods;

Supplementary Table 3 – Summary statistics of predicted absolute error of demand for family planning satisfied indicators of all inequality dimensions combined

| **Metric** | **DFPSany** | **DFPSm** |
| --- | --- | --- |
| Median absolute error | 3.2p.p. | 2.8p.p. |
| % of subgroups with absolute error < 5 p.p. | 67.1% | 76.2% |
| % of subgroups with absolute error > 10 p.p. | 9.1% | 6.0% |

DFPSany: Demand for family planning satisfied by any methods; DFPSm: Demand for family planning satisfied by modern methods; p.p: percentage points


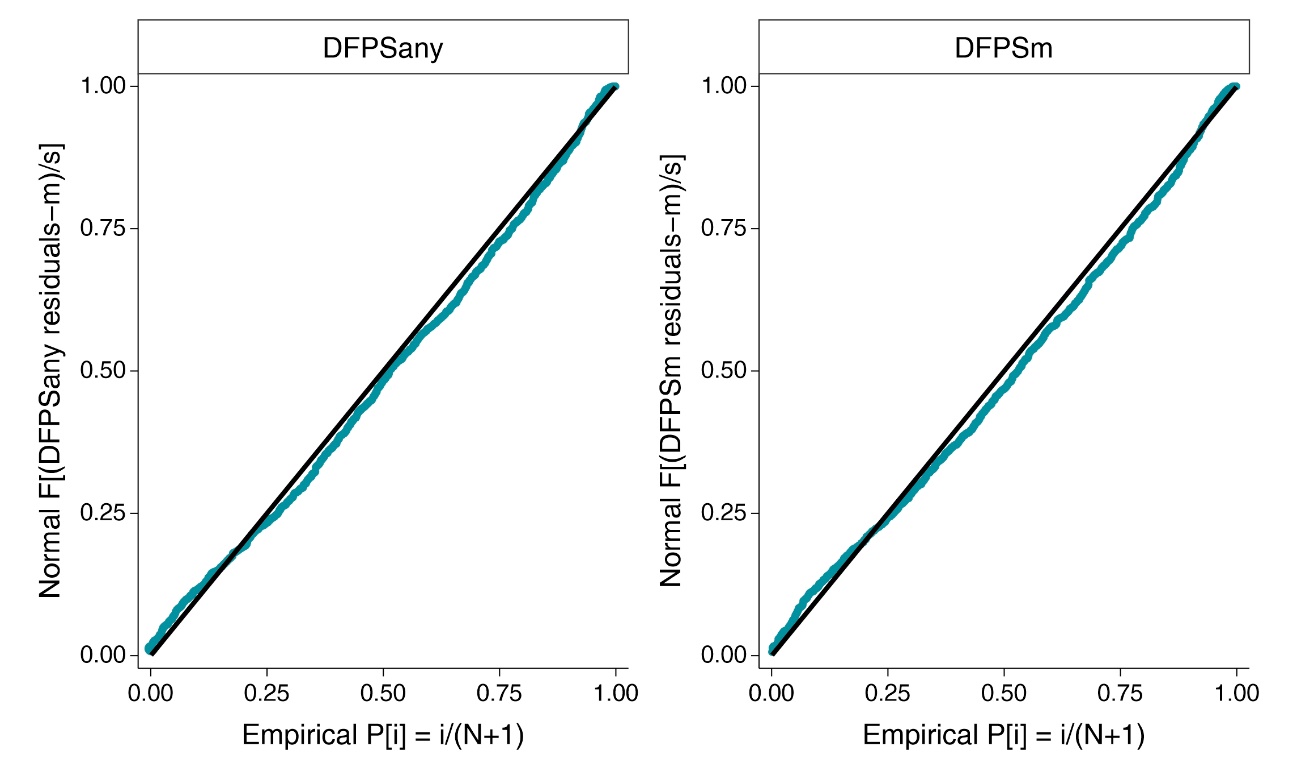


Supplementary Figure 1 – Residuals normal probability plot for DFPSm and DFPSany


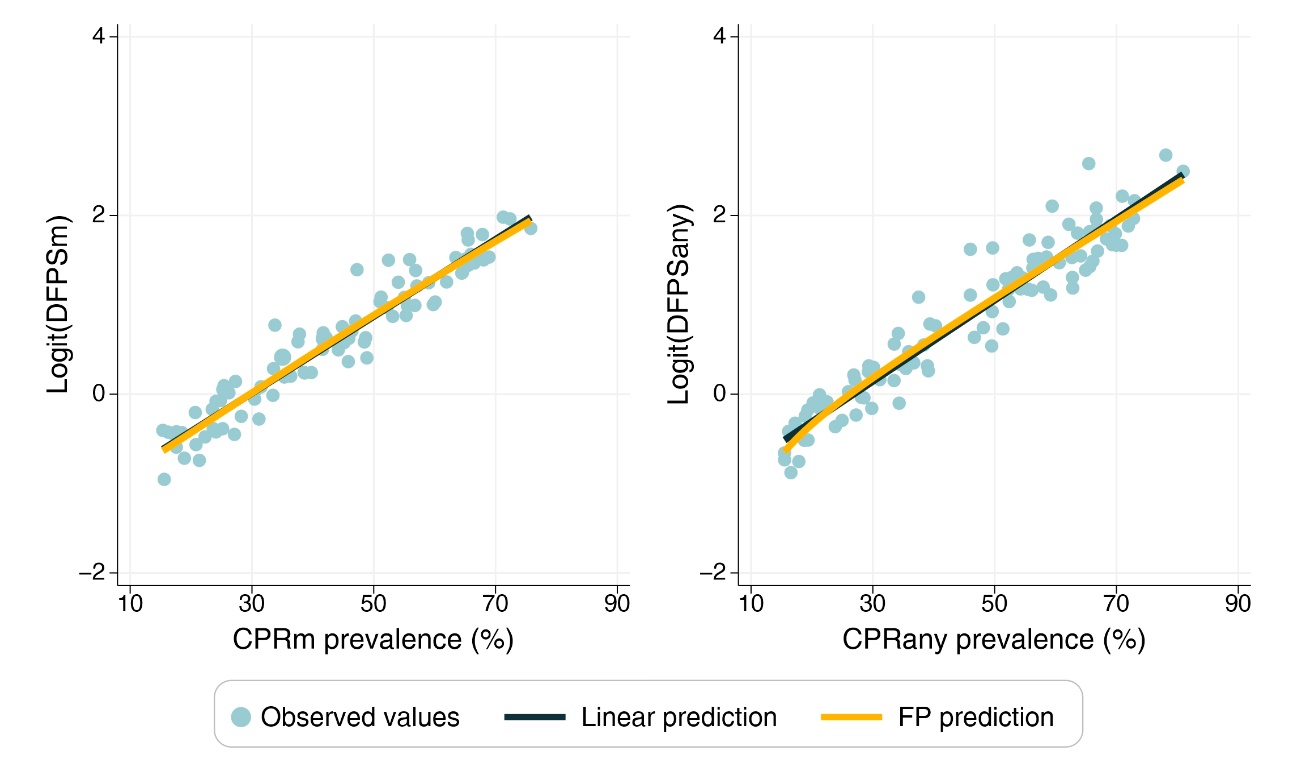


Supplementary Figure 2 – Sensitivity analysis showing the linear relationship between demand for family planning satisfied and contraceptive prevalence rate indicators where contraceptive prevalence rate is higher than 15% at national level
